# Clinically significant Prostate Cancer diagnosed using a urinary molecular biomarker-based risk score: two case reports

**DOI:** 10.1186/s12894-019-0561-6

**Published:** 2019-11-29

**Authors:** Pieter Minnee, Daphne Hessels, Jack A. Schalken, Wim Van Criekinge

**Affiliations:** 1 Department of Urology, LangeLand Ziekenhuis, Zoetermeer, The Netherlands; 2 MDxHealth BV, Nijmegen, The Netherlands; 3 Department of Urology, Radboud University Nijmegen Medical Centre, Nijmegen, The Netherlands; 4 Department of Mathematical Modelling, Statistics and Bioinformatics, Ghent University, Ghent, Belgium

**Keywords:** Case report, Risk stratification, Prostate Cancer, Risk calculator, Biopsy, Urine, Biomarkers, HOXC6, DLX1

## Abstract

**Background:**

Identifying men for a repeat prostate biopsy is a conundrum to urologists. Risk calculators (RCs) such as the European Randomized Study of Screening for Prostate Cancer (ERSPC) RCs have been developed to predict the outcome of prostate biopsies and have been shown to improve diagnostic accuracy compared to PSA alone. However, it was recently shown that the outcome for high-grade prostate cancer (PCa) upon biopsy tended to be underestimated in men with previous negative biopsies using ERSPC RC model 4. For these men, an individualized approach combining the clinical information with the outcome of biomarker-related urine tests may help to make a more informed decision.

**Case presentation:**

Two men, aged 66 and 69 respectively when presented in the clinic, show the typical dilemma of urologist and patient for electing repeat prostate biopsy. Both men had normal DRE findings, did not have a family history of PCa, presented with serum PSA values between 3 and 10 ng/ml and the first biopsies were negative for disease. The ERSPC RC4 did not indicate a biopsy in these men. The urinary molecular biomarker-based test for *HOXC6* and *DLX1*, combining biomarker-expression profiling with clinical risk factors, resulted in SelectMDx Risk scores for these men that were higher than the cut-off of the test. Based on this outcome, mpMRI was performed with an outcome of PI-RADS ≥4 in both men. Histopathological evaluation of TRUS-guided biopsies confirmed high-grade PCa.

**Conclusions:**

The urinary molecular biomarker-based risk score played a pivotal role in the diagnosis of clinically significant PCa whereas ERSPC RC4 outcome would not have indicated further diagnostic follow-up in these two cases. The timely diagnosis was shown to be crucial for the curative treatment by radical retropubic prostatectomy and the potential life-years gained for these two vital males.

## Background

Early detection of significant prostate cancer (PCa) increases the curative success rate. Histopathologic evaluation of transrectal ultrasound -guided systematic core needle biopsy (TRUS-Bx) tissue is the main method to diagnose the disease, and the decision to take a biopsy is made in case of serum prostate-specific antigen (PSA) > 3 ng/ml and/or an abnormal digital rectal examination (DRE). For more than 30 years, serum PSA has been considered the most valuable tool in early detection of the disease, but its low specificity for PCa has resulted in the detection and treatment of slow growing PCa’s that will not cause harm in a man’s lifetime and a negative biopsy rate of 70% in men with benign prostate diseases such as benign prostatic hyperplasia (BPH) or prostatitis. The chance of missing a cancer upon TRUS-Bx is ~ 25% because it is systematic, non-targeted, and directed towards the peripheral gland. Therefore, repeat biopsies are warranted in cases with persistent indication (abnormal DRE, elevated PSA value and/or histopathological findings suggestive of malignancy at the initial biopsy) [1, 2]. In the second set of biopsies, a detection rate of approximately 10–35% has been reported [2]. For a patient, these repeat biopsies will result in additional anxiety, physical discomfort and complication risk. The proportion of men undergoing biopsy who experienced non-sepsis infectious complications is ~ 18, and 3% of men experience sepsis that requires hospitalization [3, 4]. Hence a physician’s and patient’s decision to elect for prostate biopsy is guided by the fear of missing a clinically significant PCa that may become life-threatening when left untreated.

Currently, the decision to perform prostate biopsies is based on clinical judgement of the physician guided by European Association of Urology (EAU) recommendations which incorporates the results of PSA testing, DRE outcome and/or the additional diagnostic options such as a risk calculator (RC), an additional serum- or urine-based test or imaging [5].

Multiparametric MRI (mpMRI) is the most accurate imaging modality for localization of PCa. The use of mpMRI before biopsy could indicate whether the patient requires a biopsy because of a significant cancer identified on mpMRI or whether biopsy could be avoided. The EAU guideline committee recommends the use of mpMRI before repeat biopsy to allow targeted biopsies of suspicious lesions in addition to standard biopsies. However, the risk of missing 16.2 to 39.7% clinically significant prostate cancers using mpMRI targeted biopsy for mpMRI Prostate Imaging Reporting and Data System outcome (PI-RADS) ≥3 stresses the need to use RCs or biomarker-based tests for an improved risk stratification for a repeat biopsy [6].

The Rotterdam arm of the European Randomized Study of Screening for Prostate Cancer (ERSPC) developed several RCs, using the clinical data and prostate biopsy outcome from thousands of previously unscreened men and men with previous negative prostate biopsy, calculating the chance of finding PCa on a TRUS-guided biopsy. The ERSPC RC4 is designed and used by urologists to determine the likelihood of cancer in repeat biopsy. Recently, external validation of the ERSPC RC3 and ERSPC RC4 in a contemporary Dutch clinical cohort using a biopsy scheme showed that the RCs performed well, but that in the repeat biopsy setting the outcomes for PCa risk and clinically significant PCa tended to be underestimated for ERSPC RC4 [7]. For men with a previous negative biopsy, an individualized approach combining the clinical information with the outcome of biomarker-related urine tests may help to make a more informed decision.

Recently, gene expression profiling was used to identify diagnostic and predictive biomarkers for high grade PCa followed by a stepwise biomarker selection and testing of a gene panel consisting of Homeobox C6 (*HOXC6*), Tudor domain containing 1 (*TDRD1*) and Distal-less homeobox 1 (*DLX1*) in post-DRE urine sediments for the diagnosis of biopsy Gleason score ≥ 7 PCa [8]. Using whole urine as a substrate, the combination of *HOXC6* and *DLX1* had the best performance to predict high-grade PCa on biopsy which was successfully validated in an independent cohort [9]. *DLX1* and *HOXC6* are involved in prostate cancer progression and are associated with high-grade PCa [10, 11]. When this patient-specific biomarker expression profile is combined with traditional clinical risk factors, a likelihood risk score is obtained to detect clinically significant PCa (Gleason score (GS) ≥7) upon biopsy [9]. It was shown that SelectMDx risk score correlates with PI-RADS ≥3 and can contribute to the stratification of patients for mpMRI [12].

Clinical utility of this urinary molecular biomarker-based test, SelectMDx® for Prostate Cancer (hereafter SelectMDx), was evaluated in 34 men with a previous negative biopsy identified in routine clinical practice, with no study-specific visits or interventions. In five men, both the ERSPC RC4 and the SelectMDx test indicated the need for biopsy. Three men (60%) underwent prostate biopsies and in two significant PCa was found. Of the 14 men who were low risk for both the ERSPC RC4 and SelectMDx test, two (14%) underwent negative biopsies. The SelectMDx test indicated a risk for high-grade PCa upon biopsy in 15 ERSPC RC4 low risk men. Six of these men (40%) underwent prostate biopsy and in three men significant PCa was confirmed (see Table 1).

**Table 1:** Concordance table urine biomarker test versus ERSPC R4

Previous Negative Biopsy Cohort	ERSPC R4 (+)	ERSPC R4 (−)	Total
SelectMDx test (+)	5	15	20
SelectMDx test (−)	0	14	14
Total	5	29	34

This report describes two cases, identified in the latter group, in which the SelectMDx test was pivotal in the early diagnosis of clinically significant PCa.

## Case presentation 1

In January 2015, a 66-year-old male presented at the Urology ward of the LangeLand Hospital with a serum PSA of 6.4 ng/ml which was elevated compared to the upper limit of normal serum PSA of 4.5 ng/ml of men of his age. The patient had no paternal history of PCa and had no urinary complaints. The physical examination of the prostate gland (DRE) by the physician was normal. Prostate volume by TRUS was 35 cm^3^ and TRUS findings were normal. TRUS-Bx was performed (5 left and 5 right). Biopsies were negative for malignancy.

In June 2015, the serum PSA increased to 7.2 ng/ml and there was no sign of a urinary tract infection. Therefore, the physician used ERSPC RC4 with TRUS or DRE (www.prostatecancer-riskcalculator.com) containing PSA, DRE (normal/abnormal), TRUS (normal/abnormal), TRUS-assessed volume and biopsy history. This model can be used for biopsy-naïve men and for men that have had a previous biopsy with a benign result. After entering the available data, the risk of having PCa or high-grade PCa is displayed. The ERSPC RC4 showed that this man’s risk for PCa and high-grade PCa was 17 and 3%, respectively. Prostate biopsy is indicated when the ERSPC RC4 risk for PCa is > 20% and if the risk for PCa is between 12.5 and 20%, in combination with a risk for high-grade disease of > 4%. Biopsy is not indicated when the risk for PCa on biopsy is < 12.5%. Based on this outcome, patient and physician elected for PSA follow-up.

Six months later, serum PSA increased to 10.8 ng/ml. At that time the physician used the urine biomarker test, SelectMDx test (MDxHealth B.V.). After DRE, 16 ml of first void urine was collected from the patient using the urine sample collection kit (Catalogue number UrNCSE1, MDxHealth B.V.). Using this kit, the urinary RNA was immediately preserved in 4 ml of preservative. On the day of collection, samples were shipped at room temperature to the clinical diagnostic laboratory (Nijmegen, the Netherlands), after which the samples were stored at − 20 °C prior to analysis. Reverse transcriptase PCR (RT-PCR) was used to determine the amount of *HOXC6* and *DLX1* mRNA in the patient’s urine. The patient-specific biomarker expression profile was then combined with the patient’s traditional clinical risk factors, including PSA, DRE, prostate volume, age and family history using a dedicated algorithm [9]. The outcome is a patient-specific SelectMDx Risk score. If the SelectMDx Risk Score is below the cut-off point of − 2.8 a very low risk report will be generated with a negative predictive value (NPV) of 98% for Gleason score ≥ 7 PCa. A Risk Score higher or equal to this cut-off point is converted into the likelihood that subsequent biopsy will detect prostate cancer or high-grade prostate cancer. For this man, the likelihood for having PCa was 49% and the chance of having high-grade disease was 22%.

**Fig. 1:**
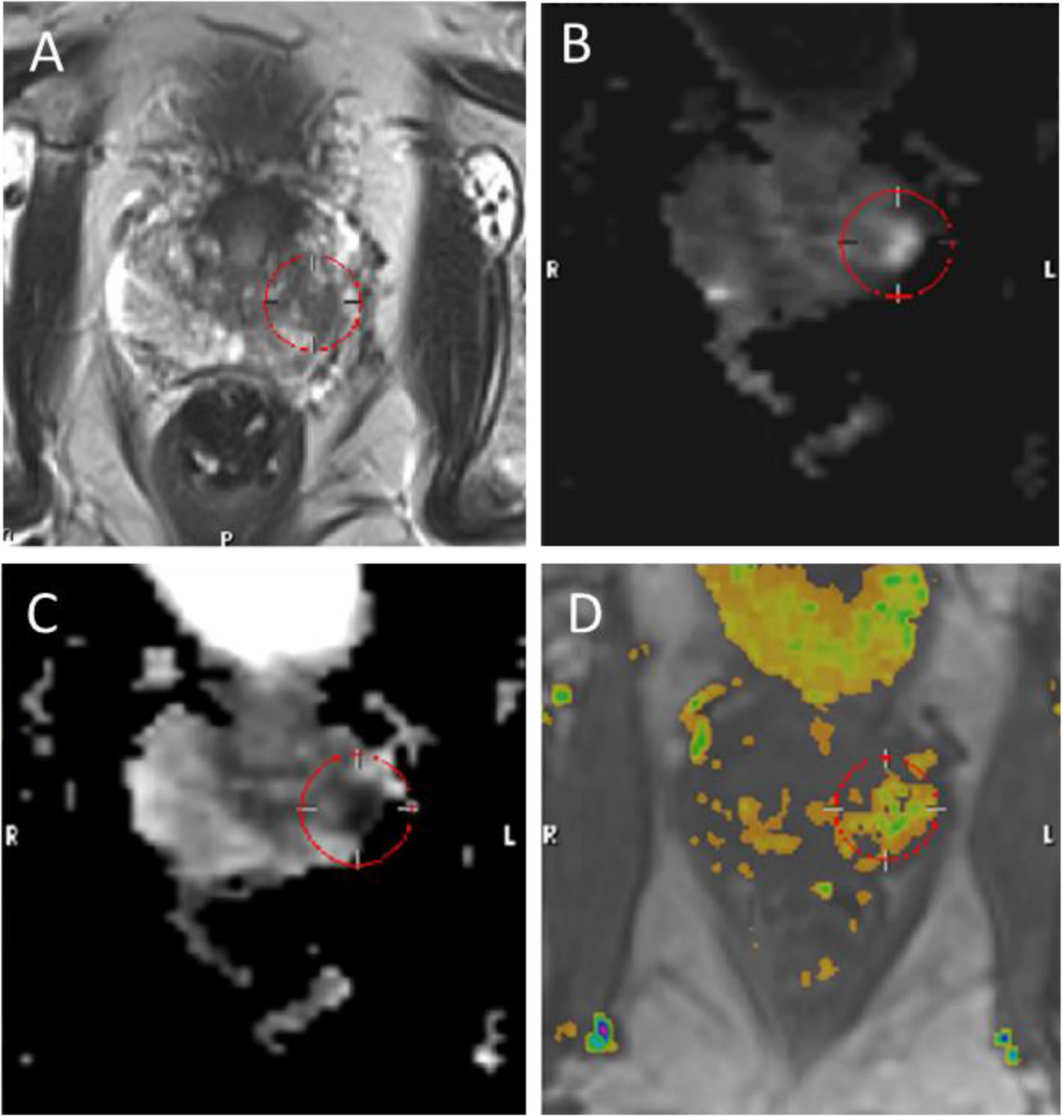
In November 2015, the patient of case 1 had a PSA level of 10.8 ng/ml and was referred for multiparametric prostate 3 T MRI for diagnostic purposes because the urinary biomarker-based risk score was 22% for high-grade PCa indicating the need for prostate biopsy. **a** An axial T2-weighted image shows a lesion on the left peripheral zone at the prostate base anterior side (red circle). **b** An axial diffusion-weighted image (DWI) shows an area of increased signal at the left peripheral zone. **c** An axial apparent diffusion coefficient (ADC) map shows a corresponding area of low ADC value (red circle). **d** A colorized perfusion map created from dynamic contrast-enhanced MRI (DCE-MRI) acquisition shows corresponding abnormal enhancement kinetics at the same location. All sequences identified the same region in the left anterior peripheral zone. The PI-RADS 5 outcome confirmed the biopsy indication of the urinary biomarker-based test outcome. TRUS-guided biopsies from that region confirmed high-grade Gleason 3 + 4 = 7 PCa

Based on this outcome, a prostate MRI was done in January 2016 (see Fig. 1). The outcome was PI-RADS 5 in the left peripheral zone of the prostate. TRUS-Bx revealed Gleason score 3 + 4 = 7 PCa in 4 of 5 biopsies taken from the left peripheral zone.

In May 2016, the patient underwent a robot-assisted radical retropubic prostatectomy (RRP). The prostate specimen was found to contain extensive carcinoma with a Gleason score 3 + 4 = 7 in both lobes (pT2c), margins and lymph nodes were clear. As part of the follow-up, the PSA is still < 0.01 ng/ml.

## Case presentation 2

In September 2012, a 69-year-old male with an unknown paternal history for PCa came to the Urology ward of the LangeLand hospital with an increased serum PSA level of 8.7 ng/ml. He had a prostate volume of 43.5 cm^3^, a normal DRE outcome and normal TRUS findings. TRUS-Bx (5 left and 5 right) were negative for malignancy. In October 2013, his PSA increased to 26.6 ng/ml. This marked elevation in serum PSA was likely caused by acute prostatitis for which the urologist prescribed antibiotics. Under antibiotics his PSA decreased to 4.3 ng/ml at the beginning of 2014.

In December 2015, the PSA was 15.4 ng/ml. The ERSPC RC4 showed that the man’s risk for PCa and high-grade PCa was 16 and 4% respectively. Based on this outcome there was no clear biopsy indication. The SelectMDx test was performed and for this man the likelihood for having PCa was 39% and the chance of having high-grade PCa was 14%. Based on this outcome, a prostate MRI was performed indicating a PI-RADS 4 in the right peripheral zone of the prostate. However, the three MRI-guided biopsies were negative for PCa.

In August 2016, the PSA increased to 20 ng/ml. Based on the prostatitis history and the negative prostate biopsies antibiotics was prescribed, but under treatment the PSA increased to 21.4 ng/ml. Since the SelectMDx test indicated an elevated risk for high-grade PCa for this patient, the urologist decided to repeat TRUS-Bx in October 2016. This resulted in the diagnosis of Gleason score 3 + 4 = 7 and 10% cribriform intraductal carcinoma in 2 out of 5 biopsies in the right peripheral zone of the prostate. The presence of intraductal carcinoma is usually associated with adverse prognostic parameters (e.g. high-grade Gleason score, large tumour volume, extra-prostatic extension and seminal vesicle invasion) and correlates with worse disease outcomes.

In December 2016, the patient underwent robot-assisted RRP. The prostate specimen was found to contain 2 tumours in the right peripheral zone of the prostate: one Gleason score 4 + 4 = 8 and one Gleason score 3 + 3 = 6 PCa (pT2a), margins and lymph nodes were clear and PSA nadir was < 0.01 ng/ml.

## Discussion and conclusions

Identifying men for a repeat prostate biopsy is a conundrum to urologists. Following negative biopsy, men frequently exhibit persistently elevated PSA, raising concerns for missed diagnosis of an aggressive PCa. Decision making regarding repeat biopsies is a balance between avoiding anxiety and risk of complications associated with the procedure and the fear for missing a clinically significant cancer. Furthermore, there is the risk with repeat biopsy of detecting and treating low-grade and low volume PCa that is not life-threatening. In this report, two cases are described that show the typical dilemma of urologist and patient within daily clinical practice for electing repeat prostate biopsy. Here the SelectMDx test played a pivotal role in the timely diagnosis of clinically significant PCa which was crucial for the curative treatment by RPP and the potential life-years gained for these vital males.

The decision to perform prostate biopsies is guided by EAU recommendations which incorporates mpMRI, RC and/or an additional serum- or urine-based test (e.g. based on *HOXC6* and *DLX1*) to individualize the need for a repeat biopsy [5]. In the second case, mpMRI targeted biopsies were performed that did not find the tumour. Recently, Pepe and collegues showed that if only targeted biopsy was performed when mpMRI showed a suspicious lesion (PI-RADS≥3), the number of biopsies could potentially have been reduced by 50%. However, 16.2–39.7% of the clinically significant prostate cancers would be missed using this approach [6]. Therefore, targeted biopsies should be done together with mapping biopsies and for risk-stratification mpMRI should be combined with an RC or biomarker-based test as the EAU guideline committee recommends.

Risk calculators are models, which take a patient’s risk factors, combines them all into an equation and assigns a level of risk for having a disease. Several RCs were developed based on the Rotterdam arm of the ERSPC, using the clinical data and prostate biopsy outcome from 3624 previously unscreened men and 2896 men with previous negative prostate biopsy [13]. For the men described in the presented cases, the physicians used ERSPC RC4 containing PSA, DRE (normal/abnormal), TRUS (normal/abnormal), TRUS-assessed volume and biopsy history. For both men, the ERSPC RC4 indicated not to perform a prostate biopsy.

Recently, in a contemporary Dutch cohort, the ERSPC PCa probability threshold of ≥20% was shown to be acceptable for the biopsy-naïve men, missing only 2% of significant cases while saving 20% of prostate biopsies [7]. However, in men with previous negative biopsies, the ERSPC RC4 underestimated the outcomes for PCa and significant PCa. At this threshold, the RC4 would have missed 23% of significant cases while saving 47% of prostate biopsies. Gayet et al. showed that a threshold of ≥10% for PCa and > 2% for significant PCa would have been most optimal for men with previous negative biopsies, missing only 2% of significant cases and saving 21% of prostate biopsies. A possible factor attributing to the underestimation could be the fact that men with a previous negative biopsy were a higher risk cohort compared with the ERSPC cohort in which every man with an elevated serum PSA > 3 ng/ml at repeat screening was biopsied again within 4 years after initial biopsy [7].

An improvement of RCs would be the inclusion of biomarkers that are measured in body fluids of a patient. Prostate cells, cancerous or benign, shed cellular content such as cell-free nucleic acid in the urine. Therefore, urine as liquid biopsy offers an attractive alternative for tissue biopsies due to the direct contact of the urinary flow with draining of the genitourinary organs such as the prostate [14]. By combining the patient-specific biomarker expression profile with the clinical information of the patient, the resulting individualized risk score will allow the physician and patient to make the most informed choice about undergoing or delaying a prostate biopsy. Van Neste et al. already showed that this newly developed risk score outperformed the Prostate Cancer Prevention Trial Risk Calculator (PCPTRC) resulting in an improved patient risk stratification for high-grade PCa and biopsy decision-making [9].

The two cases presented here were identified in routine clinical practice in a Dutch hospital, with no study-specific visits or interventions. The novel urine biomarker test led to the use of prostate MRI prior to prostate biopsy. A recent systematic review on the NPV of mpMRI in excluding PCa at biopsy showed that the NPV depends on the prevalence of PCa [15]. At an overall PCa prevalence of 30% the NPV of mpMRI could be 88%, but when compared to the prevalence of 40% in the Van Neste et al study, the NPV of mpMRi would decrease to 82% [15]. Recently, it was shown that there is a correlation between the urine biomarker risk score and mpMRI outcomes [12]. Since the NPV of the urine-based genetic test is 98% and the risk score correlates with mpMRI outcomes, this urine biomarker test may become an important decision tool, not just for biopsy, but to risk stratify patients for mpMRI as well.

Recently, Cucchiara et al. showed that genomic biomarkers in combination with clinical and pathological variables have improved the detection, prognosis, and risk evaluation of PCa [16]. The SelectMDx test is a useful tool to reduce the number of unnecessary biopsies and stratify low-risk from high-risk tumors. Cost effectiveness studies have shown that quality-adjusted survival can be improved using this test by decreasing the number of biopsies performed and by not treating indolent tumors [17–19]. We acknowledge that the here described data only represents two cases and that no firm claims can be made based on these results. Large-scale case-controlled studies should confirm the applicability and reliability of using the SelectMDx test over the ERSPC RC4 in risk stratification for repeat biopsy.

The case reports on two men with persistent suspicion of PCa but negative prostate biopsy stress the need to use all the tools that EAU guidelines propose (mpMRI, RCs, biomarker-based tests) to individualize the need for biopsy. Although RCs are recommended and commonly used by urologists to predict repeat biopsy outcome, the fact that there may be an underestimate of the outcomes for high-grade PCa stresses the need to use multivariate risk stratification tools (e.g. combine the clinical information with biomarkers for high-grade disease) in order to make a more informed repeat biopsy decision. These two routine clinical practice examples demonstrate that the urine biomarker test for *HOXC6* and *DLX1*, combining clinical data with biomarkers detected in urine from a patient, offers an easy and individualized tool for stratifying men for mpMRI and biopsy.

## Data Availability

Not applicable

## Abbreviations

ADC: Apparent Diffusion Coefficient
BPH: Benign Prostatic Hyperplasia
DCE-MRI: Dynamic Contrast-Enhanced MRI
DLX1: Distal-less homeobox 1
DRE: Digital Rectal Examination
DWI: diffusion-weighted image
EAU: European Association of Urology
ERSPC: European Randomized Study of Screening for Prostate Cancer
GS: Gleason Score
HOXC6: Homeobox C6
NPV: Negative Predictive Value
PCa: Prostate Cancer
PI-RADS: Prostate Imaging Reporting and Data System
PSA: Prostate Specific Antigen
RC: Risk Calculator
RC4: Risk Calculator Model 4
RRP: radical retropubic prostatectomy
TDRD1: Tudor domain containing 1
TRUS-Bx: Transrectal ultrasound guided biopsies
